# Nomogram to Predict Tumor-Infiltrating Lymphocytes in Breast Cancer Patients

**DOI:** 10.3389/fmolb.2021.761163

**Published:** 2021-11-26

**Authors:** Jikun Feng, Jianxia Li, Xinjian Huang, Jiarong Yi, Haoming Wu, Xuxiazi Zou, Wenjing Zhong, Xi Wang

**Affiliations:** ^1^ Department of Breast Oncology, Sun Yat-Sen University Cancer Center, The State Key Laboratory of Oncology in South China, Collaborative Innovation Center for Cancer Medicine, Guangzhou, China; ^2^ Department of Medical Oncology, The Sixth Affiliated Hospital of Sun Yat-Sen University, Guangdong Provincial Key Laboratory of Colorectal and Pelvic Floor Diseases, Guangzhou, China

**Keywords:** breast cancer, tumor-infiltrating lymphocytes (TILs), nomogram, neoadjuvant therapy (NAC), precise medicine

## Abstract

**Background:** Tumor-infiltrating lymphocytes (TILs) play important roles in the prediction of prognosis and neoadjuvant therapy (NAT) efficacy in breast cancer (BRCA) patients, in this study, we identified clinicopathological factors related to BRCA TILs, then to construct and validate nomogram to predict high density of TILs.

**Methods:** A total of 826 patients diagnosed with BRCA in Sun Yat-Sen University cancer center were enrolled in nomogram cohort. TILs were assessed using hematoxylin-eosin (H&E) staining by two pathologists. Complete clinical data were collected for analysis. Then the enrolled patients were split into a training set and validation set at a ratio of 8:2. and the backward multivariate binary logistic regression model was used to establish nomogram for predicting BRCA TILs, which were further evaluated and validated using the C-index, receiver operating characteristic (ROC) curves and calibration curves. Then another independent NAT cohort of 106 patients was established for verifying this nomogram in NAT efficacy prediction.

**Results:** TILs were significantly correlated with body mass index (BMI), tumor differentiation, ER, PR, HER2 expression, Ki67, blood biochemical indicators including total bilirubin (TBIL), indirect bilirubin (IBIL), total protein (TP), Globulin (GLOB), inorganic phosphorus (IP), calcium (Ca). In which ER expression level [OR = 0.987, 95%CI (0.982–0.992), *p* < 0.001], IP [OR = 4.462, 95%CI (1.171∼17.289), *p* = 0.029], IBIL [OR = 0.906, 95%CI (0.845–0.966), *p* = 0.004] and TP [OR = 1.053, 95%CI (1.010–1.098, *p* = 0.016)] were independent predictors of TILs. Then nomogram was established, for which calibration curves (C-index = 0.759) and ROC curve (AUC = 0.759, 95%CI 0.717–0.801) in training sets, calibration curves (C-index = 0.708) and ROC curve (AUC = 0.708, 95%CI 0.617–0.800) in validation sets demonstrated great evaluation efficiency. Besides, independent NAT cohort verified this nomogram can distinguish patients with greater NAT efficacy (*p* = 0.041).

**Conclusion:** The finds of clinicopathological factors associated with TILs could help clinicians to understand the tumor immunity of BRCA and improve treatment system for patients, and the established nomogram with high evaluation efficiency may be used as a complement tool for distinguishing patients with better NAT efficacy.

## Introduction

According to the latest cancer statistics, in 2021, there would be about 284,200 newly diagnosed breast cancer (BRCA) patients in the United States, accounting for around 15% of all cancer diagnosis. In the female population, BRCA has the highest incidence rate (30%) and the second mortality rate (15%), and the incidence has steadily increased in the past 2 decades, therefore, BRCA is considered as a major threat to women’s health (Siegel et al., 2021). Although the treatment methods of BRCA patients has been improved to a certain extent, there is still space for improvement in precise treatment and management. And in clinical practice, there is an urgent need for indicators to predict prognosis, treatment efficacy and guide selections of treatment options.

Tumor-infiltrating lymphocytes (TILs) refer to mononuclear immune cells infiltrating into the tumor tissue, which have been reported and studied in a variety of solid tumors, including BRCA, colon cancer, cervical cancer, melanoma, and lung cancer (Underwood, 1974). TILs in the breast tissue mainly consist of CD8^+^ T cells, as well as helper (CD4^+^) T cells, CD19^+^ B cells and a very small amount of NK cells with different infiltration degrees (Chin et al., 1992; Whitford et al., 1992). TILs is a highly complicated system (Gajewski et al., 2013). Thus, we need to do furthering research to better understanding tumor immunity.

TILs are of great significance in neoadjuvant treatment (NAT) efficacy evaluation for BRCA patients. Studies have shown that TILs are associated with positive disease-free survival (DFS) in triple-negative breast cancer (TNBC) and HER2 + patients. While TILs are related to increased overall survival (OS) in TNBC and HR+ HER2-patients (Denkert et al., 2018a), and TILs can serve as an independent prognosis factor for TNBC patients (Pruneri et al., 2016a). Besides, the level of TILs is also related to tumor metastasis in BRCA, research indicate metastatic BRCA tended to have lower TILs (Zhu et al., 2019). What’s more, TILs were also reported to be correlated with the efficacy of NAT. For TNBC and HR+ HER2-subtypes, patients with higher TILs are more likely to have pathological complete response (pCR) after neoadjuvant chemotherapy (Hamy et al., 2019). A possible explanation for this association was that chemotherapy, including cyclophosphamide, and gemcitabine, can indirectly stimulate TILs mediated adaptive immune response by reducing immunosuppressive factors (Denkert et al., 2018a). Therefore, chemotherapy, besides being tumoricidal, may additionally have an immunotherapeutic effect via stimulation of immune responses in TILs leading to complete clinical responses (Pruneri et al., 2016a). For HER2+ subtype, patients with higher TILs tended to benefit more from neoadjuvant targeted therapy in regard of pCR (Ignatiadis et al., 2019; De Angelis et al., 2020).And the same trend has also been observed in BRCA immunotherapy (Loi et al., 2021), However, beneficial effects of neoadjuvant endocrine-therapy were found in tumors with low TILs infiltration for HR + HER2-patients (Skriver et al., 2020a; Skriver et al., 2020b; Lundgren et al., 2020).

Overall, TILs in BRCA patients are of great clinical significance in NAT and prognosis evaluation. However, there are some limitations for traditional H and E staining. Firstly, for patients receiving NAT, tumor sample could only be obtained through pre-operative fine-needle aspiration biopsy, the sample size is small and local, which on the one hand cannot represent the whole tumor, due to the heterogeneous distribution of TILs in BRCA patients (Dieci et al., 2018), and will affect the accuracy of detection on the other hand. Therefore, a prediction model, based on accurate TILs data of NAT naive surgical resection specimens and baseline available indictors, has important clinical significance for evaluating the baseline level of TILs and the efficacy of NAT. Finally, the prediction model of TILs, can serve as a newly prognosis tool for BRCA patients, whose tumor samples cannot be obtained (et: samples from other hospitals could not be borrowed and used, or the tissue itself was too small for further staining) to perform H and E staining and morphological evaluation of TILs. Thus, for those two kinds of specific BRCA patients, traditional H and E staining has noteworthy deficiencies, so we need an prediction model with great accuracy.

Therefore, NAT naive surgical resection specimens were analyzed and clinical data was collected, aiming to explore associations of TILs with clinicopathological characteristics, and to propose nomogram model for predicting high TILs in BRCA patients.

## Materials and Methods

### Nomogram Cohort Patients

From June 2020 to March 2021, a total number of 826 patients’ data were collected in Sun Yat-Sen University Cancer Center. The diagnosis of invasive breast cancer was confirmed by postoperative pathology. The main inclusion criteria were: 1. 18–80 years of age; 2. eligibility for radical tumor resection after comprehensive evaluation. Exclusion criteria mainly were: 1) previous NAT, i.e., chemotherapy, radiotherapy, targeted therapy, or immunotherapy, etc; 2) secondary tumors or multifocal tumors; 3) HIV infection or other immune system diseases; 4) past use of immune agents or drugs and health care products that may affect immune function; 5) state of inflammation and infection within nearly 1 week. This study was approved by the Ethics Committee of Sun Yat-Sen University Cancer Center.

### General Information and Examination Results

Patients’ information was obtained from the breast cancer single disease research platform of Sun Yat-Sen University Cancer Center. Inclusion and exclusion criteria were described above. General patient information, histopathological findings and specific blood test results were assessed retrospectively. General patient information included age, gender, BMI, blood type, hypertension, and diabetes. Blood test results included blood routine, blood biochemical, hormone level, and tumor markers. All blood test results were obtained from the patients within 1 week before surgery. The histopathological data included postoperative paraffin pathological findings: pathological type and stage, TNM staging, degree of tumor differentiation, immunohistochemical staining results. ER positivity was defined as ER ≥ 1%. PR positivity was defined as PR ≥ 1%.

### Tumor-Infiltrating Lymphocyte Assessment

Postoperative tumor paraffin specimens from 826 patients underwent H and E staining, and two pathologists independently calculated stromal TILs density percentages; the final values of TILs were obtained through unified discussion, with the measurement standard referring to “RECOMMENDATIONS BY AN INTERNATIONAL TILs–For the supplemental versions of WORKING GROUP 2014 and 2018”,the standardized approach mainly including: 1) select tumor aera.2) define stromal area.3) scan at low magnification.4) determine type of inflammatory infiltrate.5) assess the percentage of stromal TILs (Salgado et al., 2015; Dieci et al., 2018). In this study, “10%” TILs was taken as the cut-off value; therefore, patients with >10% TILs were considered the high TILs group, and the remaining individuals constituted the low TILs group.

### Construction and Validation of Nomogram

To improve the robustness and reliability of our prediction model, the nomogram cohort were split into a training set and validation set in a random manner without replacement at a ratio of 8:2. Training set was used to construct the predicted nomogram and perform internal validation. For training cohort, baseline predictors of TILs in BRCA patients were evaluated by the univariate and multivariate binary logistic regression model. Then variables with *p* value less than 0.05 were included in the backward multivariate binary logistic regression model (Erratum: Borderud et al., 2015) to further screen the optimal prediction model with the lowest Akaike information criterion (AIC). Finally, nomogram establishment was performed based on the optimal prediction model with the R software (version 4.0.1). Bootstrapping with 80 samples was applied for internal validation of the nomogram. The performance of nomogram was assessed by Harrell’s concordance index (C-index). Calibration curves were implemented to validate the accuracy and reliability of the nomogram (Kramer and Zimmerman, 2007). In the end, model performance in predicting high TILs were quantified using the area under the curve (AUC) of the receiver operating characteristic (ROC) analysis in training set and validated in validation set, respectively. During the validation of the nomogram, the total points of each patient in the validation cohort were calculated according to the established nomogram, then ROC curve were derived using the total points as predicting parameter. All analyses and plots were performed using the following R packages: “rms”, “calibrate”, “pROC” and “nomogramEx”.

### Validation of Nomogram in NAT Cohort

In order to verify the clinical significance of TILs nomogram model, we included an independent cohort treated with NAT, to verify our nomogram model with the prediction of NAT pCR ratio. The main inclusion criteria were: 1) 18–80 years of age female patients; 2) Preoperative diagnosed as the early and intermediate stage breast cancer, and eligibility for NAT after comprehensive evaluation according to the latest NCCN BRCA guideline. Exclusion criteria mainly were: 1) previous anti-tumor therapy, i.e., surgery, chemotherapy, radiotherapy, targeted therapy, or immuno-therapy, etc; 2) secondary tumors or multifocal tumors; 3) HIV infection or other immune system diseases; 4) past use of immune agents or drugs and health care products that may affect immune function; 5) state of inflammation and infection within nearly 1 week; 6) Participated in any prospective drug clinical study. Then, a total of 106 patients’ data were collected in Sun Yat-Sen University Cancer Center from December 2017 to March 2021.Total points of each patient in the NAT cohort were calculated according to the established nomogram. NAT efficacy was calculated with Pathological complete response (pCR), pCR defined as pathological Miller-payne grade5 together with no lymph nodes metastasis. Patients were divided into predicted high and low TILs groups according to the optimal cut-off points defined by ROC analysis in nomogram training set.

### Statistical Analysis

Categorical variables were analyzed by the Chi-square test or Fisher’s exact test. Continuous variables were analyzed by non-parametric tests. Associations of clinical and pathological variables with TILs were determined. Then, the optimal cutoff value of TILs was determined by ROC curve analysis, predicting low and high-immune cell groups and further stratifying the patients into the low and high-TILs groups. All statistical tests were two-sided, and *p* < 0.05 was considered significant. Statistical analysis was performed with Programming language R (version 4.0.1, http://www.R-project.org).

## Results

### General Patient Characteristics and Histopathological Findings

**FIGURE 1:**
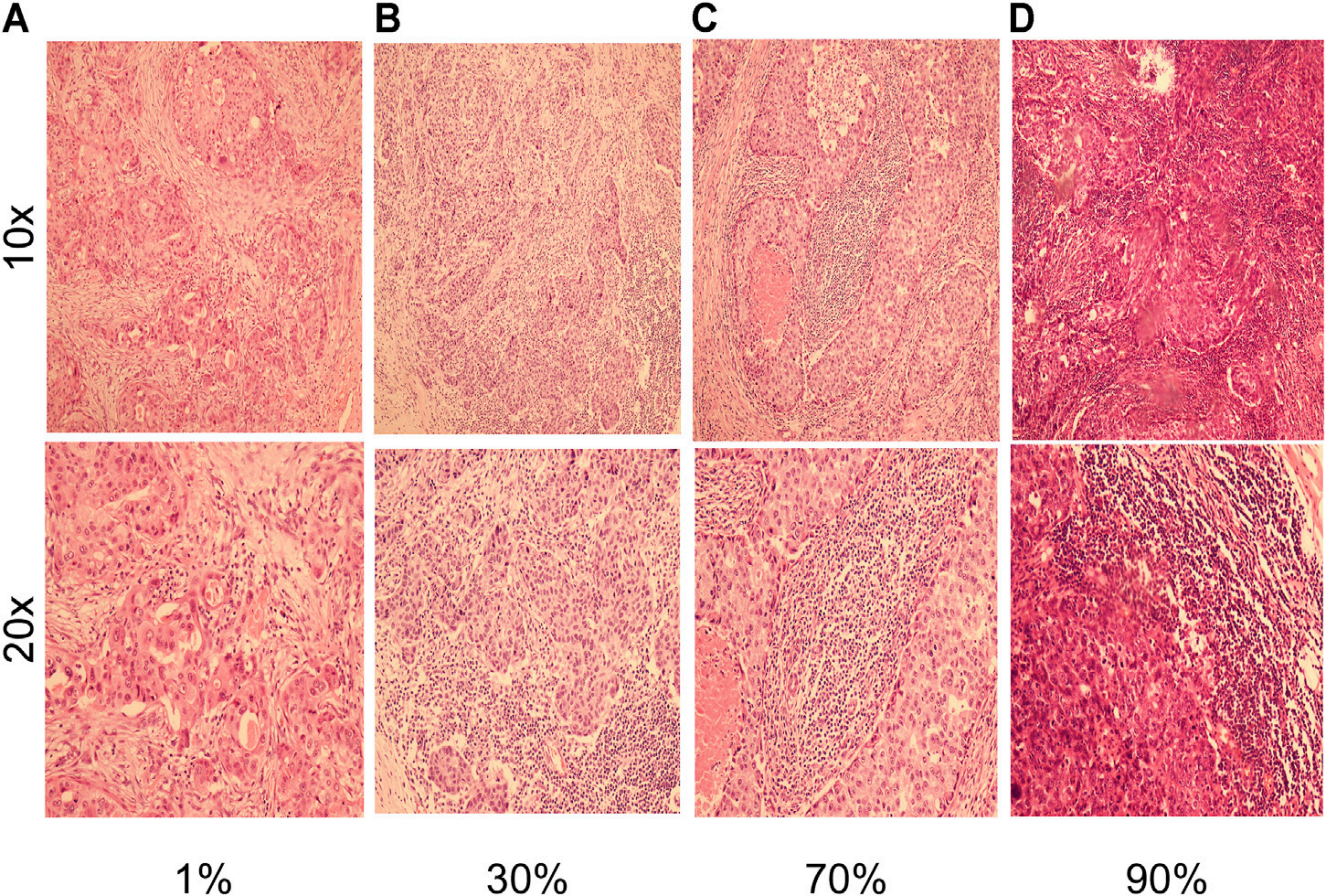
H and E staining with of breast cancer tissues, shown at 10× magnification (inset at 100×) and 20× magnification (inset at ×200). **(A)** Representative images of H and E in a breast cancer patient with 1% TILs density at 10x and 20x magnifications. **(B)** Representative images of H and E in a breast cancer patient with 30% TILs density at 10x and 20x magnifications. **(C)** Representative images of H and E in a breast cancer patient with 70% TILs density at 10x and 20x magnifications. **(D)** Representative images of H and E in a breast cancer patient with 90% TILs density at 10x and 20x magnifications.

A total of 826 patients were included in nomogram cohort according to the above criteria, H andE staining was performed in enrolled cases to assess the density of TILs, and the results were confirmed by two pathologists. As shown in Figures 1H,E, staining showed various concentration gradients of TILs under a microscope at 100 and 200×, respectively, (Figure 1). The general information is shown in Table.1. Related factors were included for analysis, and the associations of these factors with TILs in tumor tissue samples were analyzed. It was found that tumor differentiation (*p* < 0.001), TNM staging (*p* = 0.045) and BMI (*p* = 0.015) were significantly correlated with TILs. In addition, age, gender, marital status, blood group, tumor location, tumor T and N stages, hypertension, and diabetes mellitus were not significantly correlated with tumor TILs.

**TABLE 1:** Patient and general features.

	Low TILs (≤10%) *n* (%)	High TILs (>10%) *n* (%)	*p* Value
Age	—	—	0.192
≤40	84 (14)	39 (18)	—
>40	523 (86)	180 (82)	—
Sex	—	—	0.699
Female	604 (100)	219 (100)	—
Male	3 (0)	0	—
BMI	—	—	0.015
≥28	71 (12)	12 (5)	—
<28	536 (88)	207 (95)	—
Blood group	—	—	0.667
A	170 (28)	67 (31)	—
B	152 (25)	46 (21)	—
O	209 (34)	78 (26)	—
AB	42 (7)	16 (7)	—
Unknown	34	12	—
Blood Rh	—	—	1.000
+	571 (94)	206 (94)	—
-	2 (0)	1 (0)	—
Unknown	34	12	—
Location	—	—	1.000
Left side	304 (50)	109 (50)	—
Right side	303 (50)	110 (50)	—
Histology	—	—	0.648
Invasive Ductal	546 (90)	200 (91)	—
Others	61 (10)	19 (9)	—
Histological grade	—	—	<0.001
I	14 (2)	1 (0)	—
II	378 (62)	78 (37)	—
III	182 (30)	134 (61)	—
Unknown	33	6	—
N stage	—	—	0.106
N0	343 (57)	109 (50)	—
N1	155 (26)	73 (33)	—
N2	61 (10)	26 (12)	—
N3	38 (6)	10 (5)	—
Unknown	10	1	—
T stage	—	—	0.192
T1	420 (69)	145 (66)	—
T2	178 (29)	69 (32)	—
T3	5 (1)	5 (2)	—
T4	4 (1)	0	—
TNM	—	—	0.045
I	279 (46)	82 (37)	—
II	216 (36)	98 (45)	—
III	102 (17)	38 (17)	—
Unknown	10	1	—
Hypertension	—	—	0.803
No	569 (94)	207 (95)	—
Yes	38 (6)	12 (5)	—
Diabetes	—	—	0.618
No	578 (95)	211 (96)	—
Yes	29 (5)	8 (4)	—

*p < 0.05, statistically significant.

a Chi-square test; *p* < 0.05 was considered statistically significant.

Abbreviation: BMI, body mass index.

### Pathological Features

Pathological findings were collected to analyze the associations of these factors with TILs (Table.2). Comparative analysis indicated that positive status of ER (37 vs. 63%, *p* < 0.001), PR (38 vs. 62%, *p* < 0.001), AR (14 vs. 86%, *p* < 0.001) expression and peri-tumor nerve invasion (10 vs. 89%, *p* = 0.009) were correlated with lower TILs. Meanwhile, HER2 positive rate (60 vs. 40%, *p* < 0.001), Ki67 expression (*p* < 0.001), and peri-tumor vascular invasion(62 vs. 38%, *p* = 0.039) were positively correlated with high TILs.

**TABLE 2:** correlations between Pathological features and TILs.

	Low TILs (≤10%) *n* (%)	High TILs (>10%) *n* (%)	*p* Value
ER	—	—	<0.001
–	81 (13)	81 (37)	—
+	526 (87)	138 (63)	—
PR	—	—	<0.001
–	93 (15)	84 (38)	—
+	514 (85)	135 (62)	—
AR	—	—	<0.001
–	25 (4)	31 (14)	—
+	582 (96)	188 (86)	—
HER2	—	—	<0.001
–	463 (76)	131 (60)	—
+	144 (24)	88 (40)	—
Ki67	—	—	<0.001
1–25%	293 (48)	45 (21)	—
26–50%	215 (35)	80 (37)	—
51–75%	76 (13)	63 (29)	—
76–99%	23 (4)	31 (14)	—
Molecular subtype	—	—	<0.001
HR + HER2(–)	440 (72)	100 (46)	—
HER2(+)	44 (24)	88 (40)	—
TNBC	25 (4)	31 (14)	—
Peri-tumor vascular invasion	—	—	0.039
+	183 (30)	83 (38)	—
–	424 (70)	135 (62)	—
Unknown	0	1	—
Peri-tumor nerve invasion	—	—	0.009
+	109 (18)	22 (10)	—
–	498 (82)	196 (89)	—
Unknown	0	1	—

*p < 0.05, statistically significant.

aChi-square test; *p* < 0.05 was considered statistically significant.; Abbreviation: ER, estrogen receptor; PR, Progesterone receptor; AR, androgen receptor; HER2, Human epidermal growth factor receptor 2. HR + HER2-, ER+/PR+, HER2-,; HER2+, ER + -, PR + -, HER2+; TNBC, ER-, PR-, HER2-.

**FIGURE 2:**
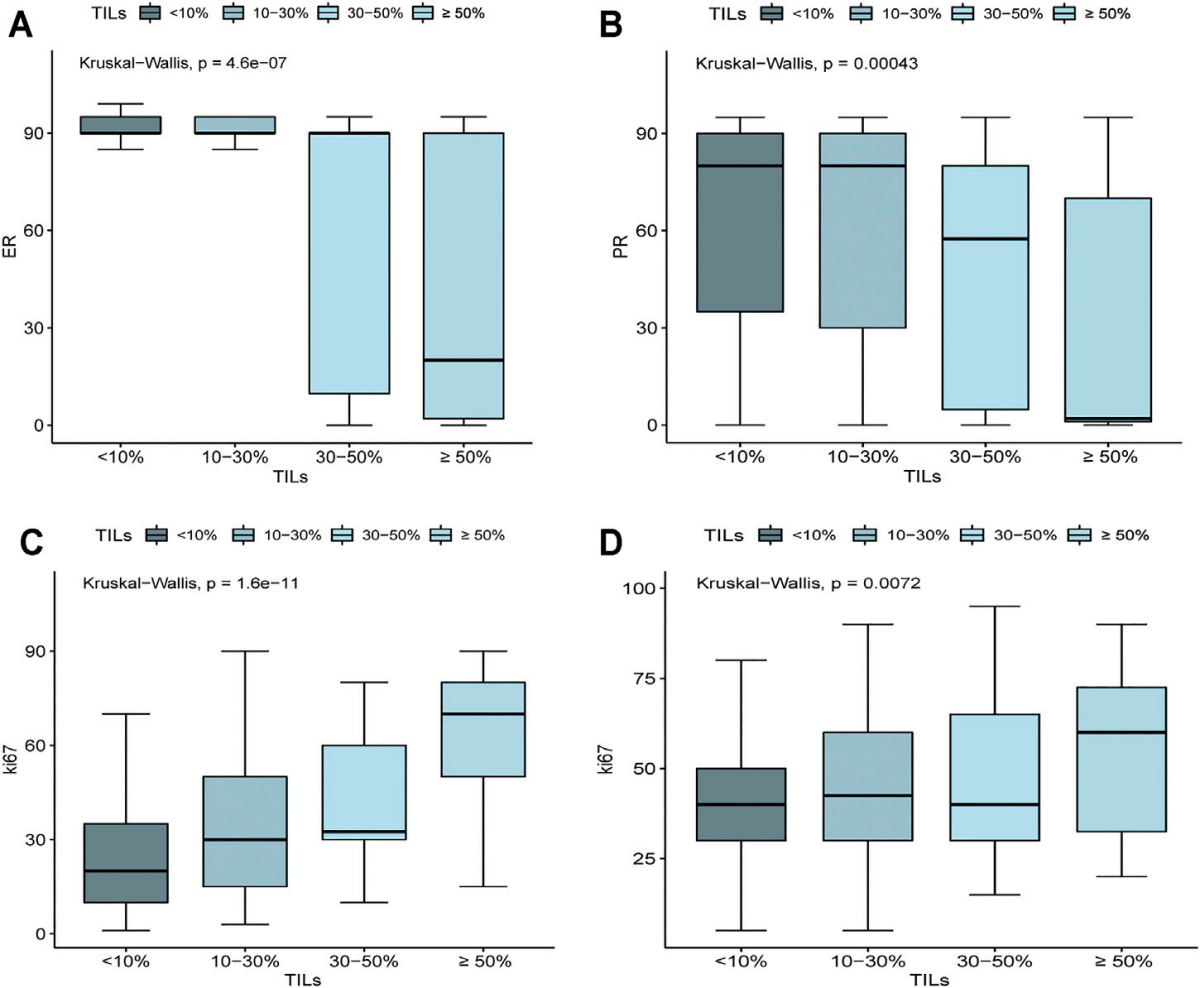
Associations of ER, PR, and Ki67 expression level with the density of TILs. **(A)** In HR+ HER2− BRCA, ER expression was negatively correlated with TILs (*p* = 4.6e-07). **(B)** In HR+ HER2− BRCA, PR expression was negatively correlated with TILs (*p* = 0.00043). **(C)** In HR+ HER2− BRCA, ki67 expression was positively correlated with TILs (*p* = 1.6e-11). **(D)** In TNBC and HER2+ BRCA, ki67 expression was positively correlated with TILs (*p* = 0.0072).

Further analysis showed that even in HR + HER2-breast cancer, ER expression level (*p* = 4.6e-07) and PR (*p* = 0.00043) were negatively correlated with TILs, indicating that the association between TILs and HR is independent of molecular subtyping. Patients with TILs <30% showed increased ER and PR expression levels, especially ER levels (Figures 2A,B). Besides, in HR + HER2-patients (*p* = 1.6e-11) and HER2+, TNBC patients (*p* = 0.0072), the expression of Ki67 was positively correlated with TILs (Figures 2C,D).

### Blood Specimen Analysis

In biochemical indicators, median levels of total bilirubin (TBIL) (10.7 vs. 10.2, *p* = 0.033) and indirect bilirubin (IBIL) (7.6 vs. 7.2, *p* = 0.018) were significantly higher in low TILs group. Conversely, median levels of total protein (TP) (72.67 vs. 73.18, *p* = 0.020), globulin (GLOB) (29.02 vs. 29.77, *p* = 0.019), calcium (Ca) (2.25 vs. 2.28, *p* = 0.011) and inorganic phosphorus (IP) (1.15 vs. 1.18, *p* = 0.006) were significantly higher in high TILs group. (Table 3).

**TABLE 3:** correlations between blood biochemistry and TILs.

	Low TILs (≤10%) N = 596 Median (Min-Max)	High TILs (>10%) N = 215 Median (Min-Max)	*p* Value
LDL.C	3.11 (1.09–6.33)	3.05 (0.90–5.43)	0.722
CHO	4.94 (2.65–8.36)	4.93 (2.05–8.62)	0.998
TBA	3.60 (0.20–137)	3.40 (0.40–51.60)	0.729
TBIL	10.7 (2.50–32.8)	10.2 (2.20–39.00)	0.033
TG	1.17 (0.38–8.51)	1.15 (0.31–8.19)	0.617
DBIL	3.10 (0.50–8.90)	2.90 (0.30–11.60)	0.148
ApoA1	1.48 (0.92–2.58)	1.49 (0.79–2.39)	0.953
ApoB	0.92 (0.40–1.86)	0.91 (0.37–1.63)	0.842
IBIL	7.60 (1.40–24.3)	7.20 (0.40–27.40)	0.018
HDL.C	1.40 (0.65–2.86)	1.37 (0.71–2.90)	0.832
LDH	158.40 (97.20–451.60)	159.40 (68.80–333.70)	0.471
UREA	4.60 (1.80–15.20)	4.60 (1.90–10.90)	0.583
UA	298.25 (94.00–609.40)	298.50 (137.10–551.00)	0.830
TP	72.67 (58.57–87.23)	73.18 (54.40–85.42)	0.020
GLOB	29.02 (20.00–42.23)	29.77 (21.30–39.38)	0.019
AG	1.50 (0.99–2.26)	1.47 (1.07–2.00)	0.110
ALB	43.45 (34.70–51.60)	43.70 (36.80–51.60)	0.403
ALP	67.30 (23.90–156.10)	67.70 (30.00–145.90)	0.382
IP	1.15 (0.77–1.75)	1.18 (0.43–1.92)	0.006
CRE	53.10 (28.80–337.80)	54.40 (36.20–117.10)	0.095
CK	65.00(19.00–578.00)	67.00 (15.00–415.00)	0.858
CHE	8,010(3,839–15,194)	8244 (4,179–15,287)	0.207
CYCS	0.82 (0.49–4.93)	0.83 (0.60–1.57)	0.374
Ca	2.25 (1.96–2.54)	2.28(1.51–2.57)	0.011
SAA	5.80 (0.50–104.60)	5.90 (0.10–380.64)	0.888
GLU	5.15 (3.98–19.05)	5.13 (4.20–11.50)	0.370
ALT	13.90 (2.90–623.40)	14.00 (1.00–70.90)	0.955
GGT	17.20 (4.60–159.90)	16.50 (6.00–857.70)	0.253
AS.AL	1.16(0.39–5.28)	1.18 (0.51–13.40)	0.421
AST	16.70 (9.20–283.10)	17.00 (9.80–66.50)	0.383

Abbreviation: LDL, very low density lipoprotein; CHO, cholesterol; TBA, total bile acid; TBIL, total bilirubin; TG, triglyceride; DBIL, direct bilirubin; ApoA1, Apolipoprotein A1; ApoB, Apolipoprotein B; IBIL, Indirect bilirubin; HDL.C, high density lipoprotein; LDH, low density lipoprotein; UREA; UA, uric acid; TP, total protein; GLOB, Globulin; AG, Anion gap; ALB, albumin; ALP, alkaline phosphatase; IP,inorganic phosphorus; CRE, creatinine; CK, Creatine kinase; CHE, cholinesterase; CYCS, Cystatin C; Ca,calcium; SAA, Serum amyloid A protein; Glu, glucose; ALT, Alanine aminotransferase; GGT, glutamyl transferase; AST, aspartate aminotransferase.

However, we found no significant correlations between TILs and indicators of blood routine (Supplementary Table S1), tumor markers (CEA, CA153, CA125, CA199) (Supplementary Table S3) and hormone levels (Supplementary Table S2).

### Nomogram for Evaluating Breast Cancer TILs

Various factors affecting BRCA TILs were included in the analysis. In order to establish a predicted model that can be used in baseline situation, we only include TILs associated factors that can be accurately detected at baseline biopsy tissue and blood samples, including ER expression level, PR expression level, Ki67 expression level, HER2 status, and significant blood indicators mentioned above (TP, GLOB, IP, Ca, TBIL, and IBLI).

**FIGURE 3:**
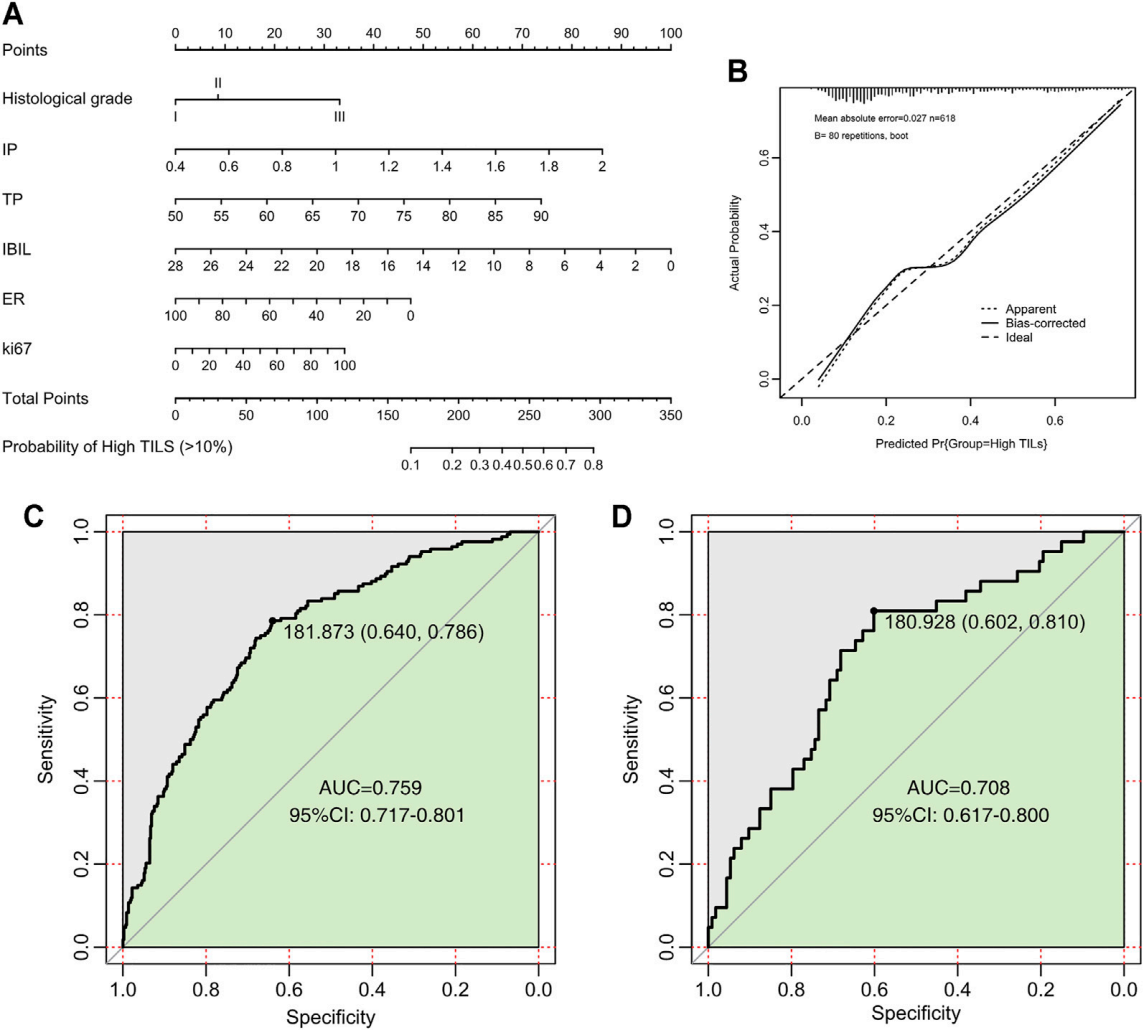
Nomogram, calibration curves, and ROC curve analysis for predicting the density of TILs in patients with breast cancer. **(A)** Prediction nomogram for TILs in breast cancer patients. **(B)** Calibration curves for predicting the density of TILs. **(C)** ROC curves for the TILs prediction nomogram in the internal training set. **(D)** ROC curves for the TILs prediction nomogram in the validation set. All the points assigned on the top point scales for various factors were summed to generate a total point score. The total score was projected on the bottom scales to determine the probability of high-density TILs in an individual. The nomogram-predicted frequency of high TIL density was plotted on the *x*-axis, and the actual observed frequency of high T cell density was plotted on the *y*-axis. The AUC was calculated, and its 95% CI was estimated by bootstrapping. TILs, tumor-infiltrating lymphocytes; ROC, receiver operating characteristic; CI, confidence interval.

We divided the nomogram cohort with complete parameter information mentioned above (*n* = 773) into training set and validation set in a random manner without replacement at a ratio of 8:2. Nomogram model construction was performed in training set (*n* = 618). In univariate logistic regression analysis, all of the baseline indicators showed significant correlation with TILs and were further enrolled in the backward multivariate logistic regression model (Table.4). It turned out that the model with IBIL, IP TP, histology grade, ER, and Ki67 as input variables has the lowest AIC. Therefore, factors of PR, HER2, TBIL, GLOB were excluded after backward multivariate binary logistic regression analysis and further nomogram construction. Finally, a nomogram model for BRCA (Figure 3) was further established with variates of IBIL, IP TP, histology grade, ER, and Ki67 (Figure 3A). Harrell’ concordance indicators of the nomogram were assessed (c-index = 0.772), and calibration curves showed good agreement between predicted and observed values (Figure 3B). In ROC curve analysis to evaluate the discrimination power of the TILs nomogram, the AUC was 0.759 (95%CI 0.717–0.801) in training set and 0.708 (95%CI 0.617–0.800) in validation set, respectively (Figures 3C,D). Univariate binary logistic analysis showed that large point value calculated using the nomogram model had a significant association with higher TILs (OR = 1.033 95%CI:1.026–1.04, *p* < 0.001).

**TABLE 4:** Correlative factors for TILs identified by univariate binary logistic regression and results of backward binary logistic multivariate logistic regression analysis.

	OR (95%CI) (univariate)	p	OR (95%CI) (multivariate)	p
BMI				
<28	1	–	—	—
≥28	0.955 (0.902–∼1.009)	0.105	—	—
Tumor differentiation				
I	1	–	1	–
II	2.163 (0.409∼39.914)	0.464	1.269 (0.231∼23.705)	0.823
III	8.326 (1.584∼153.353)	0.044	2.510 (0.437∼47.607)	0.395
ER	0.981 (0.977∼0.986)	<0.001	0.987 (0.982∼0.992)	<0.001
PR	0.983 (0.978∼0.987)	<0.001	—	—
HER2				
Negative	1	–	—	—
Positive	2.024 (1.386∼2.951)	<0.001	—	—
Ki67	1.030 (1.022∼1.038)	<0.001	1.010 (0.999∼1.020)	0.080
TBIL	0.939 (0.897∼0.981)	0.006	—	—
IBIL	0.916 (0.862∼0.97)	0.003	0.906 (0.845∼0.966)	0.004
IP	5.569 (1.65∼19.271)	0.006	4.462 (1.171∼17.289)	0.029
Ca	5.565 (0.932∼34.63)	0.063	—	—
GLOB	1.060 (1.006∼1.117)	0.028	—	—
TP	1.045 (1.006∼1.085)	0.023	1.053 (1.01∼1.098)	0.016

Abbreviation: BMI, body mass index; ER, estrogen receptor; PR, Progesterone receptor; AR, androgen receptor; HER2, Human epidermal growth factor receptor two; TBIL, total bilirubin; GLOB, Globulin; IP, inorganic phosphorus; TP, total protein; IBIL, Indirect bilirubin; Ca, calcium.

In addition, we also found that ER expression level [OR = 0.987, 95%CI (0.982–0.992), *p* < 0.001], IP [OR = 4.462, 95%CI (1.171–17.289), *p* = 0.029] ,IBIL [OR = 0.906, 95%CI (0.845–0.966), *p* = 0.004], and TP [OR = 1.053, 95%CI (1.010–1.098, *p* = 0.016)] were independent predictors of TILs in BRCA patients. (Table.4).

### Validation of Nomogram in NAT Patients

**FIGURE 4:**
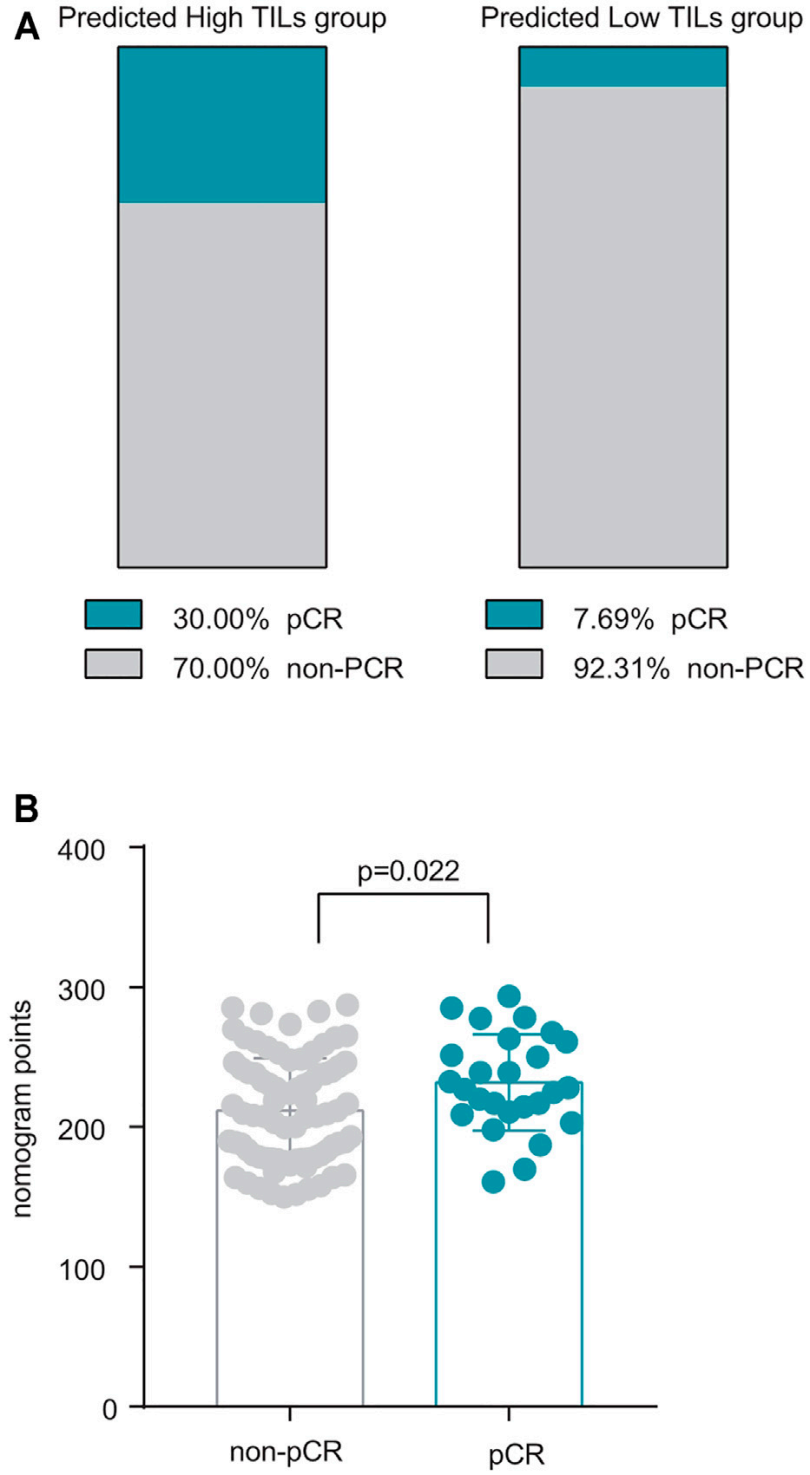
Validation of nomogram in NAT patients. **(A)** The distribution of pCR patients in the high and low TILs groups predicted by this nomogram, pCR ratio in predicted high TILs group 30 vs. 7.69% in predicted low TILs group, *p* = 0.041 **(B)** Patients in the pCR group tended to have a higher nomogram score, *p* = 0.022.

To further verify the prediction effect of this nomogram on NAT efficacy, another independent NAT cohort was collected for testing. A total of 106 patients were included, including 26 patients with pCR and 80 patients with non-PCR (Table.5), and scored those patients with this nomogram model. Patients were divided into the high TILs group and the low TILs group. 30% (24/80) of patients in the high TILs group achieved pCR, for which only 7.69% (2/26) in the low TILs group (*p* = 0.041) (Figure 4A). And in pCR group, patients tended to have higher nomogram points compared with non-pCR group (*p* = 0.022) (Figure 4B).

**TABLE 5:** general infromation in independent validation cohort.

Variables	Numbers (*n* = 106)	Percent (%)
Age(years)		
>40	83	78.3
≤40	23	21.7
Molecular subtype		
HR + HER2(-)	46	43.4
HER2 (+)	49	46.2
TNBC	11	10.4
NAT efficacy		
pCR	26	24.5
Non-pCR	80	75.5
Nomogram prediction		
high TILs group	80	75.5
low TILs group	26	24.5

Abbreviation: ER, estrogen receptor; PR, Progesterone receptor; AR, androgen receptor; HER2, Human epidermal growth factor receptor 2. HR + HER2-, ER+/PR+, HER2-,:HER2+, ER + -, PR + -, HER2+; TNBC, ER-, PR-, HER2-. pCR: Pathological complete response, TILs: tumor-infiltrating lymphocytes.

## Discussion

This study retrospectively analyzed factors affecting TILs in BRCA patients and established nomogram model with a large sample size and relatively complete and comprehensive clinical data. Through a series of statistical tests, a model with certain predictive efficacy was established and validated, and relevant factors affecting TILs were obtained. With considerable clinical significance, aiming to help clinicians understand the influencing factors of TILs and evaluate the TILs status of some specific BRCA patients. There is a nomogram model to predict TILs in ovarian cancer (Dai et al., 2021), while to our knowledge, this is the first nomogram model for predicting TILs in BRCA patients with a large sample size and explicit clinical significance.

Some influencing factors of TILs were obtained. HR + HER2- BRCA had the lowest density of TILs, significantly less than TNBC and HER2 + BRCA cases, corroborating withprevious findings (Stanton et al., 2016). In addition, based on a large sample size, this study firstly found that even in HR + HER2- BRCA, TILs were negatively correlated with ER and PR expression, and positively associated with Ki67. In TNBC and HER2+ BRCA cases, there was a positive correlation between TILs and Ki67 expression. These findings indicated that molecular subtyping of BRCA alone is not enough to predict TILs, and detailed information has valuable significance to improve TILs evaluation.

Based on the postoperative pathological results of patients in this study, TILs were correlated with pathological grade, peri-tumor vascular invasion, molecular typing and other factors, which were in agreement with the conclusions of previous studies (Pruneri et al., 2016b; Criscitiello et al., 2020; Dülgar et al., 2020). In addition, this study innovatively found that BRCA peri-tumor nerve invasion was also negatively correlated with TILs, providing another influencing factor as a reference, although the specific mechanism still needs to be further explored.

Furthermore, this study firstly found significantly negative correlations between TBIL, IBIL, and IP with TILs, based on large sample size. To our knowledge, this is the first work to report an association between bile acid metabolism and BRCA TILs. Previous studies have suggested that bile acids, as tumor suppressants, can regulate the production and function of CD4, Th17, and Treg cells in peripheral blood, which impacts the body’s tumor immunity (Hang et al., 2019; Campbell et al., 2020). Lithocholic acid (LAC) as a part of bile acid metabolism, which has been reported to inhibit the proliferation and invasion of BRCA cells and induce their death through lipid metabolism and other mechanisms (Krishnamurthy et al., 2008; Luu et al., 2018; Mikó et al., 2018). Besides, studies have shown that bile acids can affect the expression of chemokine CXCL16, and then affect the infiltration of natural killer T cells in liver cancer through intestines-liver axis, and then affect the biological behavior of tumor (Ma et al., 2018). While there is no such concept as intestines-liver axis in BRCA, and the specific mechanism behind this phenomenon remains unclear, our results may bridge a connection between bile acid metabolism, tumor immunity, and tumor biological behavior, providing clinical evidence for subsequent mechanism studies. As for IP, this study suggested a significant positive correlation with TILs. In the above multivariate logistic model, IP was the strongest independent predictor. According to previously reported data, IP increase can reduce the occurrence risk of HR- BRCA (Papadimitriou et al., 2021). Besides, some clinical BRCA treatment drugs, such as fulvestrant and bisphosphonates, may also affect IP metabolism (Robertson et al., 2016). Therefore, the relationship between IP and TILs deserves further investigation, as well as the role of IP in BRCA. Due to the limitations of retrospective study, a higher level of evidence can’t be provided, which could only confirm the correlation between the two, but not for determining the causal relationships. Furthermore, during clinical treatment, especially during NAT, abnormal blood index as bile acid or blood biochemical caused by drug therapy (as neoadjuvant chemotherapy, targeted therapy and immune-checkpoint inhibitors) or other reasons, should be paid fully attention by clinicians, which may be related with TILs and furthering tumor immunity, or even prognosis and treatment efficacy.

In recent years, the correlation between body metabolism and tumor biological behaviors has become one of the research hotspots and attracted increasing attention. This study suggested that patients’ BMI was correlated with BRCA TILs. Previous studies have reported that BMI in BRCA patients is related to tumor prognosis and sensitivity to chemotherapy: obesity increases the risk of BRCA recurrence and mortality by about 35–40% (Jiralerspong and Goodwin, 2016). Another retrospective study confirmed that BMI is associated with the efficacy of docetaxel chemotherapy in BRCA patients (Desmedt et al., 2020). Accordingly, *in vivo* experiments have confirmed that obesity can affect the function and number of CD8^+^ immune cells through the related STAT3 pathway, metabolite GATM and ACSBG1 gene (Ringel et al., 2020; Zhang et al., 2020; Maguire et al., 2021). Meanwhile this study based on lager sample size, firstly demonstrated BMI as an independent influencing factor for BRCA TILs, which may further impact on the prognosis and treatment efficacy. Consequently, the relationships and mechanism between BMI, TILs, BRCA prognosis, and treatment efficacy are worthy of further investigation. While the present findings could help us to understand the correlation between BMI and BRCA, and promote the positive effect of weight control and physical exercise on the prognosis and treatment of BRCA patients.

Studies have shown that TILs can predict the prognosis of BRCA, and can serve as an independent prognosis factor for TNBC (Pruneri et al., 2016b; Denkert et al., 2018a). Thus, the nomogram could be of great significant in prognostic evaluation especially for patients whose pathological slides cannot be obtained for further immunohistochemical staining. In addition to prognosis evaluation, studies have shown that TILs can be used as a predictor of the efficacy of different neoadjuvant therapies, a meta-analysis based on a large sample size indicated that for BRCA of various subtypes, patients with TILs >10% (intermediate and high TILs) had high NAT pCR ratio than patients with TILs ≤ 10 (Low TILs) (Denkert et al., 2018b). Besides, with the application of neoadjuvant immune-checkpoint inhibitors (ICIs) in breast cancer, Loi, S, et al. found that TILs with 10% cut-off value can be used to screen specific benefit groups of ICIs (Loi et al., 2021). Therefore, for this Nomogram model, TILs = 10% was used as cut-off value and patients were divided into two groups: patients with TILs> 10% were defined as high TILs subgroup, while TILs ≤ 10% is low TILs subgroup. Patients in the High TILs group tended to have better NAT efficacy and ICIs efficacy. And our independent NAT cohort also verified this view.

For patients receiving NAT, the method of obtaining specimens for immunohistochemical examination was fine needle biopsy. Due to the heterogeneous distribution of BRCA TILs (Denkert et al., 2018a; Dieci et al., 2018), the accuracy of TILs evaluation from needle biopsy samples were much inferior to those from postoperative specimens, which may further affected the screening of patients benefiting from NAT. In this study, we proposed a nomogram predicted model for TILs, which only required parameters that can be accurately obtained from the baseline tissue. Therefore, this nomogram model could be helpful for the evaluation of NAT efficacy. And with consideration of side effects of various treatment methods available, we could make the preferable selection for BRCA NAT.

At present, clinical evaluation methods for the efficacy of BRCA chemotherapy mainly include Oncotype DX 21 gene testing (Wang et al., 2018) and MammaPrint 70 gene testing (Krop et al., 2017). It can help clinicians to screen out suitable patients with early and middle stage HR+ HER2− BRCA, who could benefit from chemotherapy. However, those methods are expensive, and require additional blood samples from patients. While our nomogram model is convenient, quick, and non-invasive, and it can be expected to be applied in the evaluation of much more kinds of neoadjuvant therapy for subtypes of BRCA patients.

In this study, postoperative specimens were collected retrospectively for TILs assay, and factors influencing TILs were analyzed, for which may have great clinical significance. Then a nomogram model was established using basic and widely available clinical information (BMI, tumor differentiation, ER, Ki67, IP, IBIL, GLOB), which can accurately estimate the actual TILs of patients, providing a tool for patients and clinicians to make estimation of prognosis and therapeutic efficacy, so as to achieve precise treatment in BRCA.

The limitations of this study mainly include: 1) This study was a retrospective study conducted by single center, which has inherent limitations. 2) We only validated this nomogram with NAT patients in cohort with small sample size, and other items as prognosis and immunotherapy were not verified. 3) The correlation between some indicators such as bilirubin and TILs has been found, but the mechanism has not been further explored.

## Conclusion

The findings of clinicopathological factors associated with TILs could help clinicians understand the tumor immunity of BRCA patients, serval factors including IBIL and IP as independent predictors of TILs have not been reported before, which may have great clinical significance. And the established nomogram with high evaluation efficiency may be used as a complement tool for distinguishing patients with better neoadjuvant therapeutic efficacy.

## Data Availability

The original contributions presented in the study are included in the article/Supplementary Material, further inquiries can be directed to the corresponding author.
