# Supplementary material for: Nomogram to Predict Tumor-Infiltrating Lymphocytes in Breast Cancer Patients
Source: Front Mol Biosci. 2021 Nov 26;8:761163. doi: 10.3389/fmolb.2021.761163 (PMC8662984; doi:10.3389/fmolb.2021.761163)
Supplement: Supplementary file 1 [file DataSheet1.docx]

**Supplementary Materials:** The following are available online at

|  | Low TILs（≤ 10%）  N=607  Median（Min-Max） | High TILs（> 10%）  N=219  Median（Min-Max） | **P value** |
| --- | --- | --- | --- |
| WBC | 6.01(3.1-42.55) | 6.02(2.99-15.38) | 0.853 |
| NE% | 63.0(32.6-97.30) | 62.20(38.30-87.80) | 0.693 |
| NE | 3.71(1.48-41.40) | 3.67(1.33-13.41) | 0.979 |
| MO% | 5.50(0.4-11.70) | 5.40(2.80-10.70) | 0.882 |
| MO | 0.33(0.14-0.87) | 0.33(0.16-0.80) | 0.911 |
| BA% | 0.50(0.1-2.20) | 0.50(0.10-1.80) | 0.368 |
| BA | 4.00(1-13.00) | 4.00(2.00-12.00) | 0.607 |
| EO% | 1.50(0-16.10) | 1.40(0.10-7.90) | 0.562 |
| EO | 0.08(0-1.53) | 0.08(0.01-0.49) | 0.532 |
| LY% | 28.60(2.00-59.40) | 29.10(6.50-54.00) | 0.442 |
| LY | 1.69(0.31-4.10) | 1.77(0.72-3.74) | 0.467 |
| MCV | 90.60(56.50-109.20) | 90.60(55.40-100.80) | 0.404 |
| MCH | 29.80(15.90-36.90) | 30.00(14.70-34.40) | 0.609 |
| MCHC | 327.00(240.00-366.00) | 326.00(266.00-362.00) | 0.917 |
| HCT | 39.80(25.40-51.30) | 40.00(27.20-47.50) | 0.331 |
| RDW.CV | 12.60(11.00-24.70) | 12.40(10.60-24.30) | 0.108 |
| RDW.SD | 41.50(32.00-59.80) | 41.40(34.00-55.80) | 0.660 |

**Table.S1** correlations between blood routine and TILs

Abbreviation: WBC, white blood cell count; NE, neutrophil; MO, monocyte ratio; BA, basophil; EO, eo sinophil; LY, lymphocyte; MCV, mean corpuscular volume; MCH, mean corpuscular hemoglobin; MCHC, mean corpuscular hemoglobin concentration; HCT, hematocrit; RDW.CV, coefficient variation of red blood cell volume distribution; RDW.SD, standard deviation in red cell distribution width.

|  | Low TILs（≤ 10%）  N=453  Median（Min-Max）/N（%） | High TILs（> 10%）  N=151  Median（Min-Max） /N（%） | P value |
| --- | --- | --- | --- |
| E2 |  |  | 0.570 |
| ≤110 | 22(5) | 5(3) |  |
| > 110 | 431(95) | 146(97) |  |
| FSH |  |  | 0.422 |
| ≥40 | 256(57) | 79(52) |  |
| < 40 | 197(43) | 72(48) |  |
| LH | 14.86(0.227-95.18) | 11.45(1.02-88.6) | 0.860 |
| PRL | 316.9(85.16-1828) | 340.2(38.15-1509) | 0.382 |
| PROG | 0.724(0.16-93.05) | 0.98(0.164-100.1) | 0.135 |
| TESTO | 0.868(0.09-29.52) | 0.875(0.09-2.16) | 0.902 |

**Table.S2** correlations between hormonal readiness and TILs

Abbreviation: E2，Estradiol；FSH, follicle-stimulating hormone; LH, Luteinizing hormone；PRL, prolactin；PROG，progesterone；TESTO, testosterone.

|  | Low TILs（≤ 10%）  N=533  N（%） | High TILs（> 10%）  N=190  N（%） | P value |
| --- | --- | --- | --- |
| CEA |  |  | 0.779 |
| ≤5 | 503(94) | 181(95) |  |
| >5 | 30(6) | 9(5) |  |
| CA153 |  |  | 1.000 |
| ≤28 | 501(94) | 178(94) |  |
| >28 | 32(6) | 12(6) |  |
| CA125 |  |  | 0.404 |
| ≤35 | 497(93) | 173(91) |  |
| >35 | 36(7) | 17(9) |  |
| CA199 |  |  | 0.928 |
| ≤37 | 516(97) | 183(96) |  |
| >37 | 17(3) | 7(4) |  |

**Table.S3** correlations between tumor marker and TILs

Nonparametric test; p<0.05 was considered statistically significant.

Abbreviation: CEA, carcinoembryonic antigen，CA153, carbohydrate antigen 153; CA125, carbohydrate antigen 125; CA199, carbohydrate antigen 199;
